# Loss of chromosome Y is unrelated to the composition of the tumor microenvironment and patient prognosis in muscle-invasive urothelial bladder cancers

**DOI:** 10.1186/s12885-025-14019-w

**Published:** 2025-04-14

**Authors:** Henning Plage, Viktoria Ahlburg, Sebastian Hofbauer, Kira Furlano, Sarah Weinberger, Annika Fendler, Florian Roßner, Simon Schallenberg, Sefer Elezkurtaj, Niclas C. Blessin, Maximilian Lennartz, Andreas H. Marx, Henrik Samtleben, Margit Fisch, Michael Rink, Marcin Slojewski, Krystian Kaczmarek, Thorsten Ecke, Stefan Koch, Nico Adamini, Ronald Simon, Guido Sauter, Henrik Zecha, Joachim Weischenfeldt, Tobias Klatte, Sarah Minner, David Horst, Thorsten Schlomm, Martina Kluth

**Affiliations:** 1 Department of Urology, Charité – Universitätsmedizin , Corporate Member of Freie Universität Berlin, Humboldt-Universität Zu Berlin, Berlin Institute of Health, Berlin, Germany; 2 Institute of Pathology, University Medical Center Hamburg-Eppendorf, Martinistr. 52, Hamburg, 20246 Germany; 3 Institute of Pathology, Charité – Universitätsmedizin , Corporate Member of Freie Universität Berlin, Humboldt-Universität Zu Berlin, Berlin Institute of Health, Berlin, Germany; 4 Department of Pathology, Academic Hospital Fuerth, Fuerth, Germany; 5 Department of Urology, University Medical Center Hamburg-Eppendorf, Hamburg, Germany; 6 Department of Urology, Marien Hospital Hamburg, Hamburg, Germany; 7 Department of Urology and Urological Oncology, Pomeranian Medical University, Szczecin, Poland; 8 Department of Urology, Helios Hospital Bad Saarow, Bad Saarow, Germany; 9 Department of Pathology, Helios Hospital Bad Saarow, Bad Saarow, Germany; 10 Department of Urology, Albertinen Hospital, Hamburg, Germany; 11 Biotech Research & Innovation Center (BRIC), University of Copenhagen, Copenhagen, Denmark

**Keywords:** Loss of chromosome Y, Tissue microarray, Urothelial bladder cancer, Tumor microenvironment

## Abstract

**Background:**

Loss of chromosome Y (LOY) has recently been proposed to be associated with cancer aggressiveness, altered T-cell function, and poor prognosis in bladder carcinomas.

**Methods:**

Chromosome Y was analyzed using fluorescence *in-situ* hybridization on a tissue microarray containing 2,071 urothelial carcinomas of the urinary bladder from male patients, including 487 patients who had undergone cystectomy for muscle-invasive disease and for whom follow-up data were available. Data on tumor microenvironment were obtained from a previous study.

**Results:**

LOY was found in 26.0% of 1,704 analyzable cancers. In non-invasive cancers, LOY frequency was comparable in pTa G2 (22.8%) and pTa G3 (24.1%, *p* = 0.8036) carcinomas and slightly increased from pTa to pT2 - 4 carcinomas (23.1% for pTa and 27.2% for pT2 - 4) but these differences were not significant (*p* = 0.0794). In muscle-invasive cancers, LOY frequency slightly increased from pT2 (25.5%) to pT4 cancers (33.0%), but this association was not significant (*p* = 0.1814). Among pT2 - 4 cancers, LOY was associated with venous invasion (*p* = 0.0010) but unrelated to pT, pN, and L-status, as well as to overall, recurrence-free, and cancer-specific survival. Muscle-invasive urothelial carcinomas with and without LOY did not show significant differences in the number of CD8 positive lymphocytes, fraction of CD8 positive intraepithelial lymphocytes, number of macrophages and dendritic cells, and fraction of T helper and T regulatory cells.

**Conclusion:**

The lack of a clear association of LOY with histopathological parameters of cancer aggressiveness, patient prognosis, and parameters describing the tumor microenvironment strongly argues against the driving role of LOY in bladder cancer progression and cancer-associated immune reactions.

## Introduction

Sex disparities in the incidence and mortality rates of cancer are well known. For most tumor entities, men have a higher incidence and mortality rate than women, including gastrointestinal, kidney, skin, brain, bladder, and head and neck carcinomas [1]. In addition, tumor entities with a higher incidence in women, such as breast and thyroid carcinomas, usually have a better survival rate than these tumor entities in men [1]. The reasons for these sex disparities have been extensively examined but are still not fully understood. For example, male tumors often have a higher mutation burden than female tumors [2–4]. The higher mutation burden may be explained by sex differences in the efficiency of the mismatch repair system in some tumor entities, including stomach or esophageal carcinomas [4]. However, there is also evidence that sex chromosomes influence tumor development and progression (summarized in [5]).

The Y chromosome includes various genes with a male-specific function, but also genes that are necessary for the regulation of gene transcription, mRNA translation, and protein stability, such as sex determining region Y (*SRY*), eukaryotic translation initiation factor 1 A Y-linked (EIF1 AY), and ubiquitin-specific peptidase 9 Y-linked (USP9Y) [6]. Loss of chromosome Y (LOY) is a common age-related event in males, which may be associated with a higher risk of developing some cancer types, such as leukemia, breast, and head and neck carcinomas [7–10]. In urothelial bladder cancer, Abdel-Hafiz et al. [11] recently proposed that LOY may represent a critical cancer-driving event associated with high cancer aggressiveness, poor survival, and altered T-cell function in muscle-invasive urothelial carcinoma. However, this is in contrast with several previous studies that failed to find associations between LOY and tumor phenotype or patient prognosis in cohorts of 16 to 477 noninvasive and muscle-invasive bladder carcinomas [12–19].

This study aimed to further assess the impact of LOY on tumor aggressiveness, patient prognosis, and composition of the tumor microenvironment in urothelial bladder cancer. Therefore, we analyzed chromosome Y copy number status by fluorescence *in-situ* hybridization (FISH) on a tissue microarray (TMA) containing 2,071 urothelial carcinomas of the urinary bladder from male patients, including 487 patients with muscle-invasive disease, and follow-up data after cystectomy. All of these tumors have been extensively analyzed for their immune cell composition.

## Material and methods

### Tissue Microarrays (TMA)

The TMAs were first employed in a study of the prognostic role of GATA3 expression measured by immunohistochemistry in urothelial bladder cancer [20]. All TMAs contained one sample each from urothelial bladder tumors of 2,071 male and 639 female (control) patients archived at the Institute of Pathology, Charité Berlin, Germany Institute of Pathology, University Hospital Hamburg, Germany, Department of Pathology, Helios Hospital Bad Saarow, Germany, or Department of Pathology, Academic Hospital Fuerth, Germany, and/or treated at the Department of Urology, Charité Berlin, Germany, Department of Urology, University Hospital Hamburg, Germany, Department of Urology, Albertinen Hospital, Hamburg, Germany, Department of Urology, Helios Hospital Bad Saarow, Germany, and Department of Urology and Urological Oncology, Pomeranian Medical University, Szczecin, Poland. At each center the patients were treated according to current guidelines. Shortly, transurethral bladder tumor resection with or without postoperative instillation therapy was used for pTa tumors. Radical cystectomy was applied for all pT2-pT4 cancers in our patient cohort. Table 1 shows all available histopathological data, including grade, tumor stage (pT), status of lymphatic (L) and venous (V) invasion, as well as lymph node status (pN). For 487 male patients with pT2 - 4 carcinomas who were treated with cystectomy clinical follow-up data were available as follow: overall survival—time between cystectomy and death (median: 16 months, range: 1–176 months, 487 patients), recurrence-free survival—time between cystectomy and recurrence (median: 11 months, range: 1–74 months, 185 patients), and cancer-specific death—time between cystectomy and cancer-specific death (median: 15 months, range: 1–77 months, 185 patients). All tumor tissues were fixed in formalin (4% buffered) and embedded in paraffin. The manufacturing process for TMAs has been described in detail, before [21, 22]. Shortly, one tissue spot (diameter: 0.6 mm) per tumor (patient) was removed. Data on 53 different tumor microenvironment parameters were obtained from a previous study [23].

**Table 1:**
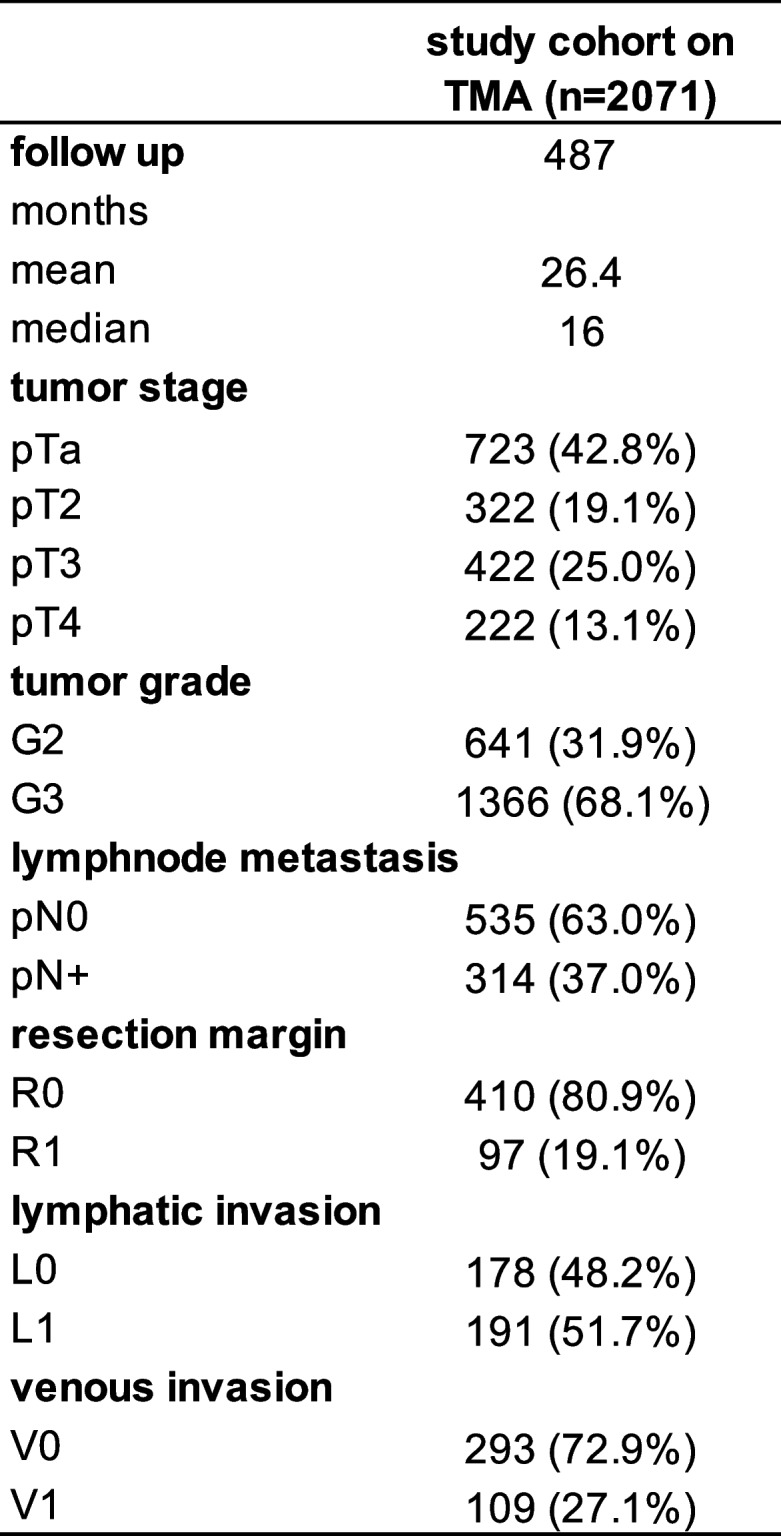
Patient cohort

Percent in the column "study cohort on TMA" refers to the fraction of samples across each category. Numbers do not always add up to 2,071 in the different categories because of cases with missing data.

### Fluorescence *in-situ* hybridization (FISH)

**Fig. 1:**
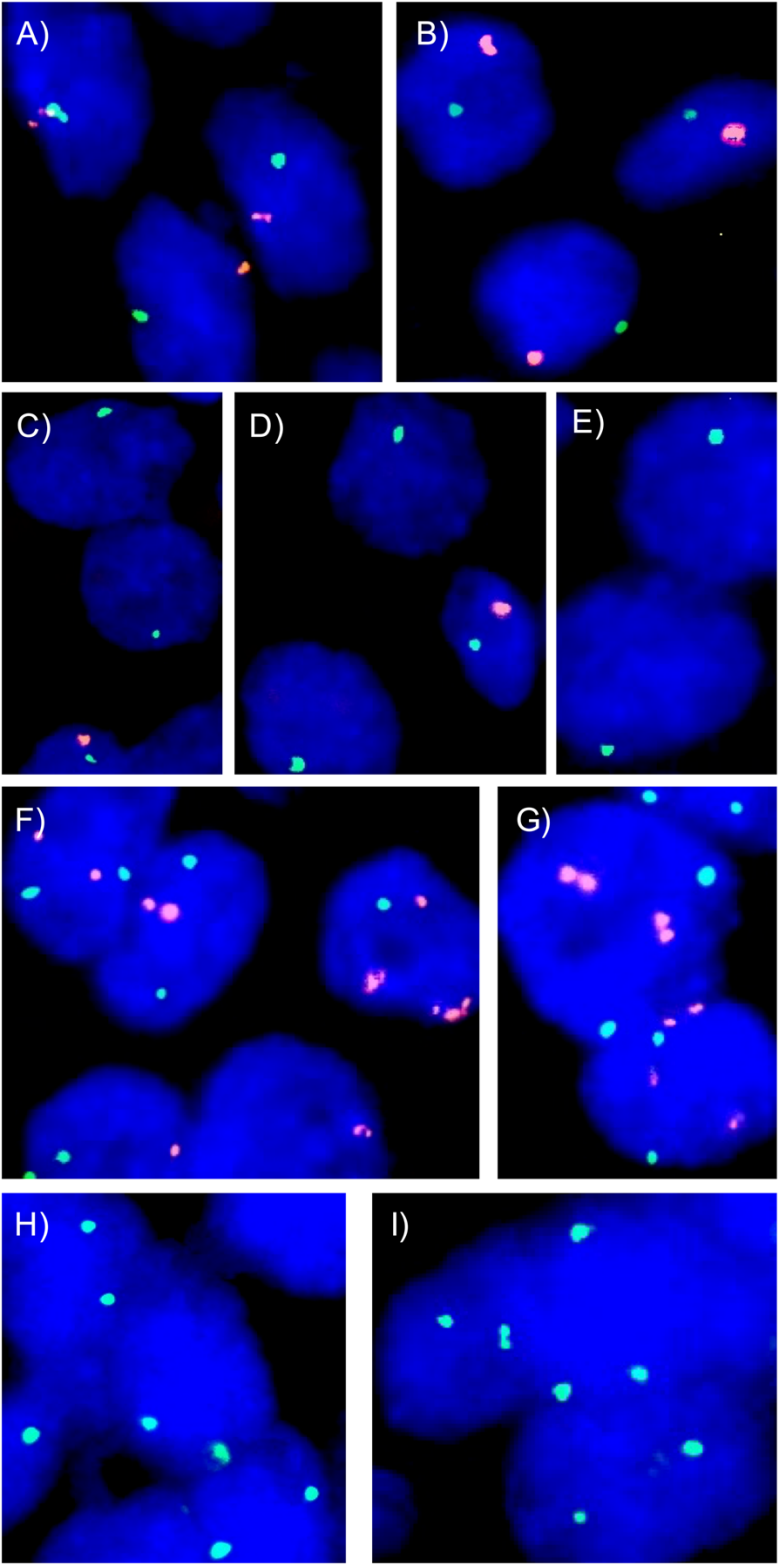
Examples of chromosome Y copy number status. A/B) chromosome Y normal with one orange chromosome Y and one green chromosome X signal, C/D/E) loss of chromosome Y without any orange chromosome Y signal in tumor cell nuclei but one orange chromosome Y signal in adjacent normal cell nuclei and green chromosome X signals in both cell nuclei types F/G) gain of chromosome Y with ≥ two orange chromosome Y signal and two chromosome X signals, H/I) normal chromosome X status of female patients with two green chromosome X signals

TMA Sections. (5 µm cut thickness) were deparaffinized with xylol, rehydrated through a graded alcohol series, and exposed to heat-induced denaturation in a water bath at 99 °C for 10 min in P1 pretreatment solution (Agilent Technologies, Santa Clara, CA, USA; #K5799). For proteolytic treatment VP2000 protease buffer (Abbott, North Chicago, IL, USA; #2 J.0730) was used and slides were incubated at 37 °C in a water bath for 200 min. A commercial FISH probe kit containing centromere Y and X probes was used for copy number detection of chromosome Y (AneuVysion Multicolor DNA Probe Kit; Abbott, Chicago, IL, USA; #05 J38). Hybridization was made overnight at 37 °C in a humidified chamber. Post-hybridization washes were carried out according to the manufacturer’s information (Agilent Technologies, Santa Clara, CA, USA; #K5799). Cell nuclei were dyed with 125 ng/ml 4’,6-diamino- 2-phenylindole in antifade solution (Biozol; Eching, Germany; #VEC-H- 1200). Using an epifluorescence microscope, stained cell nuclei were manually interpreted and copy numbers of chromosomes Y and X were estimated for each TMA tissue core, as described before [13]. Absence of chromosome Y signal in all tumor cell nuclei but presence of one chromosome Y signal in normal cell nuclei and of at least one chromosome X signal in tumor and normal cell nuclei was considered loss of chromosome Y (LOY). Presence of two or more chromosome Y signals in at least 60% of all tumor nuclei and presence of one chromosome Y signal in normal cell nuclei were considered as gain of chromosome Y (GOY). The presence of one chromosome Y and one chromosome X signal in the tumor cell nuclei was considered as chromosome Y normal. For statistical analysis, tumor spots with GOY and normal chromosome Y status were summarized as non-deleted chromosome Y. The tumor spots of female patients were used for successful hybridization of the TMA slide. TMA spots without any detectable chromosome Y signal in all tumor and normal cells were excluded from the analysis because of the lack of an internal control for successful hybridization. Figure 1 shows examples of tumors with different Y chromosome copy number statuses.

### Statistics

For statistical calculations JMP17® software (SAS®, Cary, NC, USA) was used. Chi-square test and contingency tables were used to search for associations between the copy number status of chromosome Y and tumor phenotype. The Kaplan–Meier method was used to calculate survival curves. Significant differences between the groups were estimated by the Log-Rank test.

## Results

### Technical issues

A total of 1,704 of 2,071 (82.3%) urothelial carcinoma tissue spots from male patients were informative in our chromosome Y FISH analysis. Reasons for non-informative cases (367 spots; 17.7%) included lack of tissue spots, insufficient hybridization with absence of chromosome Y and X signals in cancer and non-neoplastic nuclei, or absence of unequivocal cancer nuclei in the TMA spot.

### Loss of chromosome Y (LOY) in urothelial carcinomas

**Fig. 2:**
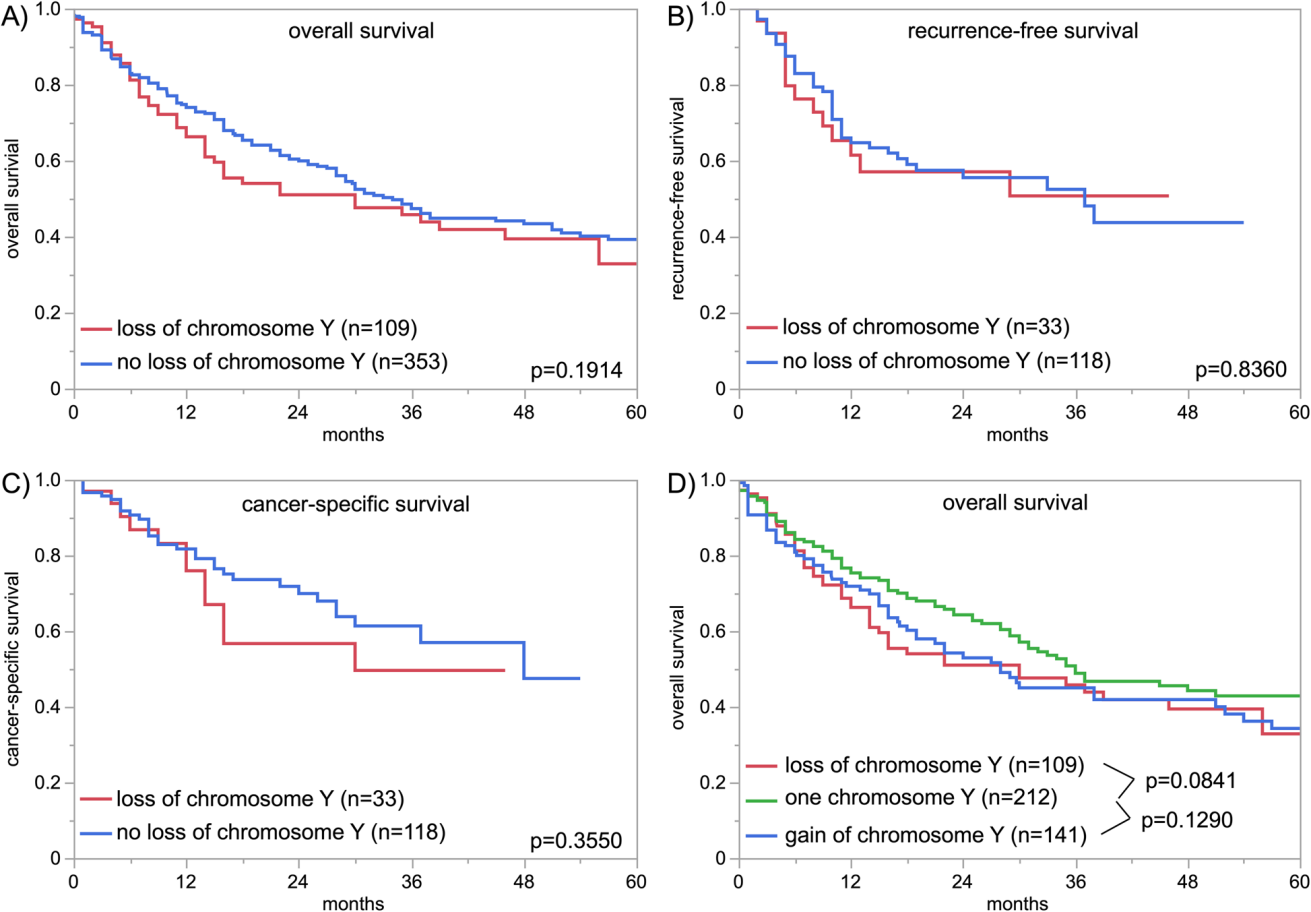
Loss of chromosome Y and patient prognosis.** A**) loss of chromosome Y versus normal status of chromosome Y and overall survival, **B**) loss of chromosome Y versus normal status of chromosome Y and recurrence-free survival, **C**) loss of chromosome Y versus normal status of chromosome Y and cancer-specific survival, and **D**) loss of chromosome Y versus one chromosome Y versus gain of chromosome Y and overall survival

LOY was detectable in 436 (25.6%) and GOY was detected in 471 (27.6%) of the 1,704 analyzable carcinomas. The fraction of LOY cancers was comparable in pTa G2 (22.8%) and pTa G3 (24.1%, *p* = 0.8036) carcinomas and slightly increased from pTa to pT2 - 4 carcinomas (23.1% for pTa and 27.2% for pT2 - 4) but these differences were not significant (0.0794; Table 2). In addition, there is no statistical difference between the frequency of LOY in pTa G3 (24.1%) and pT2 - 4 (27.2%; *p* = 0.5391) carcinomas. In muscle-invasive cancers, the frequency of LOY increased also slightly from pT2 (25.5%) to pT4 cancers (33.0%), but this association was again not significant (*p* = 0.1814). In pT2 - 4 carcinomas, the frequency of LOY was associated only with V1 (*p* = 0.0010). No association was found between LOY and grade, pN, and L-status (Table 2), or with overall, recurrence-free, and cancer-specific survival (Fig. 2). Comparable results were obtained when tumors with GOY were separated, and tumors with LOY and normal chromosome Y copy number status (*n* = 1) were compared. In these analyses, the frequency of LOY was associated only with pT (*p* = 0.0097), L1 (*p* = 0.0001), and V1 (*p* = 0.0200; Table 3, Fig. 2D).

**Table 2:**
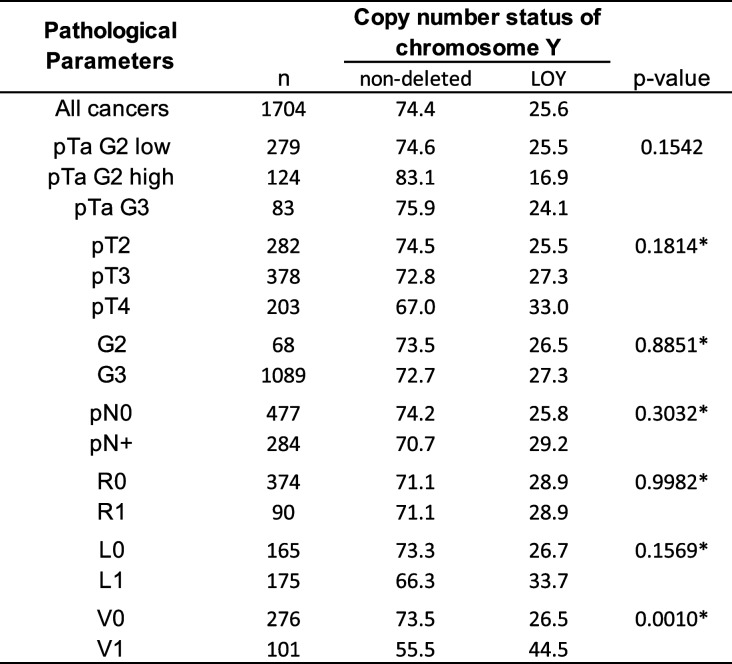
Loss of chromosome Y and tumor phenotype

*Abbreviations*: *LOY* loss of chromosome Y, *pT* pathological tumor stage, *G* Grade, *pN* pathological lymph node status, *R* resection margin status, *L* lymphatic invasion, *V* venous invasion, *only in pT2-4 urothelial carcinoma

**Table 3:**
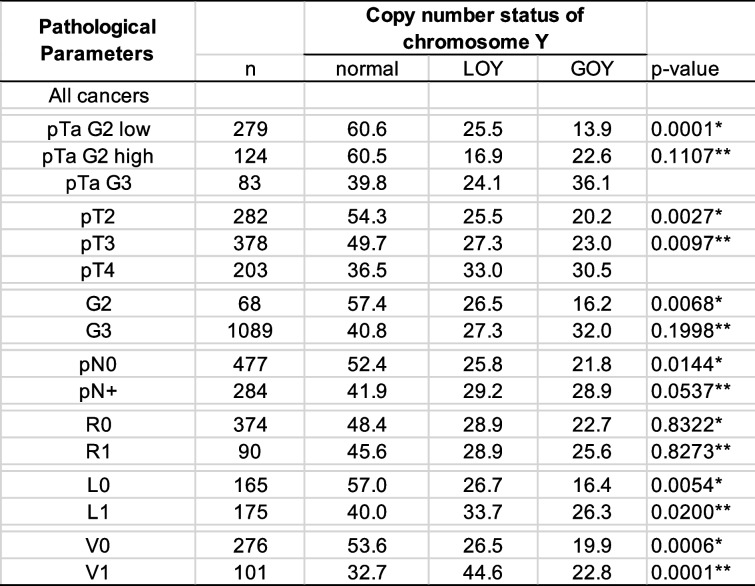
Loss and gain of chromosome Y and tumor phenotype

*Abbreviations*: *LOY* loss of chromosome Y, *GOY* gain of chromosome Y, *pT* pathological tumor stage,* G* Grade, *pN* pathological lymph node status, *R* resection margin status, *L* lymphatic invasion, *V* venous invasion, *normal, LOY, and GOY, **normal versus LOY

### LOY and tumor microenvironment

**Fig. 3:**
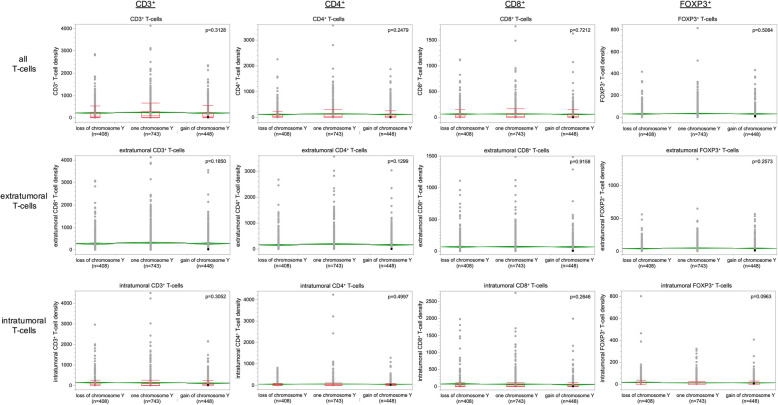
Loss of chromosome Y and tumor microenvironement. The chromosome Y status was compared with the expression of CD3, CD4, CD8 and FOXP3 in all T-cells, extratumoral T-cells and intratumoral T-cells

Results on chromosome Y status were compared with parameters of the tumor microenvironment in 1,599 carcinomas from a previous study. In these analyses, LOY was not associated with the amount of CD3, CD4, CD8, and FOXP3 positivity on all, extra-tumoral, or intra-tumoral T-Cells in the tumor microenvironment (Fig. 3).

## Discussion

In this study, we did not find a significant relationship between LOY and parameters of cancer aggressiveness, patient prognosis, or the composition of the tumor microenvironment of muscle-invasive urothelial bladder cancers.

LOY was found in 26% of 1,704 analyzable urothelial carcinomas in our TMA FISH analysis. This frequency is in the range of previous FISH studies showing LOY in 22% to 41% (mean: 26%) of 16 to 477 analyzed urothelial bladder carcinomas [12–19]. The fact that these data from different studies are rather similar is probably due to the high reproducibility of FISH data. FISH is the gold standard method for analyzing gene and chromosome copy numbers in tissues composed of mixed cell types, because a reliable morphology-based cell-by-cell analysis of the contained DNA copies can be executed. Therefore, most chromosomal Y studies have used FISH to determine the frequency of LOY in bladder carcinomas. A few studies evaluating DNA or RNA from disaggregated tissues to study Y chromosome copy numbers have found more variable and partly higher rates of Y chromosome loss ranging from 22 to 61% [11, 24].

LOY was only marginally related to some parameters of tumor aggressiveness in our analysis of 1,704 carcinomas. The slight increase in LOY from pTa G2 to pT4 cancers is consistent with a continuous loss of dispensable Y chromosomes during tumor evolution over time. The absence of significant associations between LOY and important clinicopathological parameters such as pT, pN, and survival within pT2 - 4 and the lack of statistical difference between the LOY frequency in pTa G3 and pT2 - 4 cancers argues against a significant cancer-promoting role of LOY in urothelial carcinoma. Our largely negative results are in line with data from previous FISH studies, which were also unable to find significant associations between LOY and overall survival in muscle-invasive and recurrence-free survival in non-muscle-invasive bladder cancer [13, 16, 18]. LOY determined by FISH was also unrelated to unfavorable tumor features or poor patient outcomes in cohorts of 1,045 renal cell carcinomas [25], 2,053 prostate adenocarcinomas [26], 30 esophageal squamous cell carcinomas [27], and 35 hepatocellular carcinomas [28]. Overall, these data do not provide any evidence for a particular cancer-driving role for Y chromosome loss in cancer.

These data are in conflict with recently published data by Abdel-Hafiz et al., who proposed a marked prognostic role of LOY based on the analysis of the TCGA dataset by using a sophisticated algorithm depending on the expression of 18 Y chromosome genes, which resulted in a very high rate of 61% LOY cases. While this approach only resulted in a marginal prognostic difference (*p* = 0.029) in a cohort of 300 muscle-invasive urothelial carcinomas from the TCGA database, the authors further corroborated the notion of a cancer-driving role of LOY by a two-fold higher tumor growth rate for Y^−^ cells compared to Y^+^ cells in immunocompetent mice. As a potential explanation for the differences between Y^−^ and Y^+^ cells, the authors described a markedly suppressed immune response in seven mice with Y^−^ tumors compared to 10 Y^+^ mice. In addition, Y^−^ cells in these mice responded better to anti-PD- 1 immunotherapy than Y^+^ cells in mice in vitro [11]*.*

Because of these data by Abdel-Hafiz et al., we were interested in the relationship between the tumor microenvironment and LOY in our patients. Using the same TMAs as in this study, we recently analyzed the prognostic role of the tumor microenvironment by multicolor immunohistochemistry using 21 different antibodies [23]. The delineation of 53 different immune cell phenotypes and their spatial relationship led to the identification of 32 immune parameters that were linked to prolonged overall survival (p ≤ 0.003), 15 of which were independent of pT and pN stage. The validity of our approach was also supported by the strong prognostic role (among all analyzed parameters) of CD8^+^ T cells, which were in direct contact with tumor cells. The extinction of tumor cells by cytotoxic T cells constitutes the intuitive terminal end route of the antitumor immune response. None of the tumor microenvironment and immune cell parameters were associated with LOY in 1,599 male patients with muscle-invasive urothelial carcinomas, which did not support the role of LOY in the immune escape of these tumors in vivo.

However, our study has also limitations. The number of examined tumors is still too small to investigate all difference between subgroups. Particular, a higher number of cases than 487 male patients with clinical follow-up data would be preferable. In addition, the lack of information on additional cancer treatment, including information about adjuvant or neoadjuvant chemotherapy as well as the retrospective nature of our analysis are further limitations of our study.

## Conclusions

The lack of a clear association of LOY with histopathological parameters of cancer aggressiveness, patient prognosis, and parameters describing the tumor microenvironment strongly argues against the driving role of LOY in bladder cancer progression and cancer-associated immune reactions.

## Data Availability

All data generated or analyzed during this study are included in this published article.

## Abbreviations

EIF1 AY: Eukaryotic translation initiation factor 1 A Y-linked
FISH: Fluorescence *in-situ* hybridization
GOY: Gain of chromosome Y
L: Lymphatic invasion
LOY: Loss of chromosome Y
NGS: Next generation sequencing
pN: Lymph node status
pT: Tumor stage
SRY: Sex determining region Y
TCGA: The Cancer Genome Atlas
TMA: Tissue microarray
USP9Y: Ubiquitin specific peptidase 9 Y-linked
V: Venous invasion

## References

[1] Sung H, Ferlay J, Siegel RL, Laversanne M, Soerjomataram I, Jemal A, et al. Global Cancer Statistics 2020: GLOBOCAN Estimates of Incidence and Mortality Worldwide for 36 Cancers in 185 Countries. CA Cancer J Clin. 2021;71(3):209–49. 33538338 10.3322/caac.21660

[2] Xiao D, Pan H, Li F, Wu K, Zhang X, He J. Analysis of ultra-deep targeted sequencing reveals mutation burden is associated with gender and clinical outcome in lung adenocarcinoma. Oncotarget. 2016;7(16):22857–64. 27009843 10.18632/oncotarget.8213 PMC5008406

[3] Gupta S, Artomov M, Goggins W, Daly M, Tsao H. Gender Disparity and Mutation Burden in Metastatic Melanoma. J Natl Cancer Inst. 2015;107(11). 10.1093/jnci/djv221 PMC4643631 26296643

[4] Li CH, Haider S, Shiah YJ, Thai K, Boutros PC. Sex Differences in Cancer Driver Genes and Biomarkers. Cancer Res. 2018;78(19):5527–37. 30275052 10.1158/0008-5472.CAN-18-0362

[5] Lopes-Ramos CM, Quackenbush J, DeMeo DL. Genome-Wide Sex and Gender Differences in Cancer. Front Oncol. 2020;10: 597788. 33330090 10.3389/fonc.2020.597788 PMC7719817

[6] Bellott DW, Hughes JF, Skaletsky H, Brown LG, Pyntikova T, Cho TJ, et al. Mammalian Y chromosomes retain widely expressed dosage-sensitive regulators. Nature. 2014;508(7497):494–9. 24759411 10.1038/nature13206 PMC4139287

[7] Forsberg LA, Rasi C, Malmqvist N, Davies H, Pasupulati S, Pakalapati G, et al. Mosaic loss of chromosome Y in peripheral blood is associated with shorter survival and higher risk of cancer. Nat Genet. 2014;46(6):624–8. 24777449 10.1038/ng.2966 PMC5536222

[8] Noveski P, Madjunkova S, Sukarova Stefanovska E, Matevska Geshkovska N, Kuzmanovska M, Dimovski A, et al. Loss of Y Chromosome in Peripheral Blood of Colorectal and Prostate Cancer Patients. PLoS ONE. 2016;11(1): e0146264. 26745889 10.1371/journal.pone.0146264 PMC4706411

[9] Hollows R, Wei W, Cazier JB, Mehanna H, Parry G, Halford G, et al. Association between loss of Y chromosome and poor prognosis in male head and neck squamous cell carcinoma. Head Neck. 2019;41(4):993–1006. 30582241 10.1002/hed.25537 PMC6492017

[10] Agahozo MC, Timmermans MAM, Sleddens H, Foekens R, Trapman-Jansen A, Schroder CP, et al. Loss of Y-Chromosome during Male Breast Carcinogenesis. Cancers (Basel). 2020;12(3). 10.3390/cancers12030631 PMC7139680 32182822

[11] Abdel-Hafiz HA, Schafer JM, Chen X, Xiao T, Gauntner TD, Li Z, et al. Y chromosome loss in cancer drives growth by evasion of adaptive immunity. Nature. 2023;619(7970):624–31. 37344596 10.1038/s41586-023-06234-x PMC10975863

[12] Khaled HM, Aly MS, Magrath IT. Loss of Y chromosome in bilharzial bladder cancer. Cancer Genet Cytogenet. 2000;117(1):32–6. 10700863 10.1016/s0165-4608(99)00126-0

[13] Minner S, Kilgue A, Stahl P, Weikert S, Rink M, Dahlem R, et al. Y chromosome loss is a frequent early event in urothelial bladder cancer. Pathology. 2010;42(4):356–9. 20438408 10.3109/00313021003767298

[14] Sauter G, Gasser TC, Moch H, Richter J, Jiang F, Albrecht R, et al. DNA aberrations in urinary bladder cancer detected by flow cytometry and FISH. Urol Res. 1997;25(Suppl 1):S37–43. 9079755 10.1007/BF00942046

[15] Sauter G, Moch H, Wagner U, Novotna H, Gasser TC, Mattarelli G, et al. Y chromosome loss detected by FISH in bladder cancer. Cancer Genet Cytogenet. 1995;82(2):163–9. 7664248 10.1016/0165-4608(95)00030-s

[16] Neuhaus M, Wagner U, Schmid U, Ackermann D, Zellweger T, Maurer R, et al. Polysomies but not Y chromosome losses have prognostic significance in pTa/pT1 urinary bladder cancer. Hum Pathol. 1999;30(1):81–6. 9923932 10.1016/s0046-8177(99)90305-2

[17] Panani AD, Roussos C. Sex chromosome abnormalities in bladder cancer: Y polysomies are linked to PT1-grade III transitional cell carcinoma. Anticancer Res. 2006;26(1A):319–23. 16475713

[18] Aly MS, Khaled HM, Emara M, Hussein TD. Cytogenetic profile of locally advanced and metastatic Schistosoma-related bladder cancer and response to chemotherapy. Cancer Genet. 2012;205(4):156–62. 22559976 10.1016/j.cancergen.2012.01.011

[19] Nemoto R, Nakamura I, Uchida K, Harada M. Numerical chromosome aberrations in bladder cancer detected by in situ hybridization. Br J Urol. 1995;75(4):470–6. 7788258 10.1111/j.1464-410x.1995.tb07267.x

[20] Plage H, Samtleben H, Hofbauer S, Kornienko K, Weinberger S, Bruch PG, et al. GATA3 expression loss is linked to stage progression but is unrelated to prognosis in muscle-invasive urothelial carcinoma of the bladder. Hum Pathol. 2022;130:10–7. 36152841 10.1016/j.humpath.2022.09.004

[21] Bubendorf L, Nocito A, Moch H, Sauter G. Tissue microarray (TMA) technology: miniaturized pathology archives for high-throughput in situ studies. J Pathol. 2001;195(1):72–9. 11568893 10.1002/path.893

[22] Kononen J, Bubendorf L, Kallioniemi A, Bärlund M, Schraml P, Leighton S, et al. Tissue microarrays for high-throughput molecular profiling of tumor specimens. Nat Med. 1998;4(7):844–7. 9662379 10.1038/nm0798-844

[23] Debatin NF, Bady E, Mandelkow T, Huang Z, Lurati MCJ, Raedler JB, et al. Prognostic Impact and Spatial Interplay of Immune Cells in Urothelial Cancer. Eur Urol. 2024;1:42–51. 10.1016/j.eururo.2024.01.023 38383257

[24] Richter J, Beffa L, Wagner U, Schraml P, Gasser TC, Moch H, et al. Patterns of chromosomal imbalances in advanced urinary bladder cancer detected by comparative genomic hybridization. Am J Pathol. 1998;153(5):1615–21. 9811354 10.1016/S0002-9440(10)65750-1 PMC1853402

[25] Buscheck F, Fraune C, Garmestani S, Simon R, Kluth M, Hube-Magg C, et al. Y-chromosome loss is frequent in male renal tumors. Ann Transl Med. 2021;9(3):209. 33708836 10.21037/atm-20-3061 PMC7940894

[26] Stahl PR, Kilgue A, Tennstedt P, Minner S, Krohn A, Simon R, et al. Y chromosome losses are exceedingly rare in prostate cancer and unrelated to patient age. Prostate. 2012;72(8):898–903. 21956681 10.1002/pros.21492

[27] Yamaki H, Sasano H, Ohashi Y, Shizawa S, Shineha R, Satomi S, et al. Alteration of X and Y chromosomes in human esophageal squamous cell carcinoma. Anticancer Res. 2001;21(2A):985–90. 11396192

[28] Aly MS, Bahnassy AA, Abdel-Rahman ZN. Investigation of chromosomal aberrations in Egyptian hepatocellular carcinoma patients by fluorescence in situ hybridization. Indian J Hum Genet. 2010;16(2):87–93. 21031057 10.4103/0971-6866.69370 PMC2955957

